# Effects of PM_10 _in human peripheral blood monocytes and J774 macrophages

**DOI:** 10.1186/1465-9921-5-29

**Published:** 2004-12-21

**Authors:** DM Brown, K Donaldson, V Stone

**Affiliations:** 1 School of Life Sciences, Napier University, Edinburgh, UK; 2 ELEGI Laboratory, Wilkie Building, University of Edinburgh, UK

**Keywords:** Macrophage, Nanoparticle, Cytokine, Cytoskeleton, PM_10_

## Abstract

The effects of PM_10_, one of the components of particulate air pollution, was investigated using human monocytes and a mouse macrophage cell line (J774). The study aimed to investigate the role of these nanoparticles on the release of the pro-inflammatory cytokine TNF-α and IL-1α gene expression. We also investigated the role of intracellular calcium signalling events and oxidative stress in control of these cytokines and the effect of the particles on the functioning of the cell cytoskeleton. We showed that there was an increase in intracellular calcium concentration in J774 cells on treatment with PM_10 _particles which could be significantly reduced with concomitant treatment with the calcium antagonists verapamil, the intracellular calcium chelator BAPTA-AM but not with the antioxidant nacystelyn or the calmodulin inhibitor W-7. In human monocytes, PM_10 _stimulated an increase in intracellular calcium which was reduced by verapamil, BAPTA-AM and nacystelyn. TNF-α release was increased with particle treatment in human monocytes and reduced by only verapamil and BAPTA-AM. IL-1α gene expression was increased with particle treatment and reduced by all of the inhibitors. There was increased F-actin staining in J774 cells after treatment with PM_10 _particles, which was significantly reduced to control levels with all the antagonists tested. The present study has shown that PM_10 _particles may exert their pro-inflammatory effects by modulating intracellular calcium signalling in macrophages leading to expression of pro-inflammatory cytokines. Impaired motility and phagocytic ability as shown by changes in the F-actin cytoskeleton is likely to play a key role in particle clearance from the lung.

## Introduction

Increased exposure to PM_10 _particles is associated with adverse health effects [1,2]. Much of the mass of PM_10 _is low in toxicity and it has been suggested that, combustion-derived nanoparticles (ultrafine particles) [3-5] are a key component that drives these effects, especially inflammation. In individuals with pre-existing lung disease, inhalation of nanoparticles may induce inflammation and exacerbate respiratory and cardiovascular effects through the induction of oxidative stress and inflammation [4,6,7]. Rat inhalation studies using nanoparticles of various types, at high exposure, have demonstrated pulmonary fibrosis, lung tumours, epithelial cell hyperplasia, inflammation and increased cytokine expression [8-11].

The alveolar macrophage plays an important role in particle-mediated inflammation by phagocytosing particles and release of pro-inflammatory mediators such as the cytokine tumor necrosis factor-alpha (TNF-α) [12]. The signalling mechanisms for transcription of the TNFα gene includes calcium-related pathways in diseases such as sepsis [13-15]. Calcium is released from the endoplasmic reticulum stores on stimulation of the cell, leading to a calcium influx across the plasma membrane via calcium channels [16]. Various pathogenic particles have been shown to produce such changes in calcium flux within the cell [17,18] and a large number of pathological responses could be stimulated via such calcium signalling.

In order for macrophages to migrate and phagocytose foreign material, an intact functional cytoskeleton is necessary. The cytoskeleton is sensitive to ROS and oxidative stress, due to the presence of thiol groups located on the actin microfilaments. On oxidation, these filaments cross-link, leading to reduced cell motility, impaired phagocytosis and hence clearance of foreign material from the lung. The cytoskeleton mediates several basic cell functions: chemotaxis, migration, phagocytosis, phagosome-lysosome fusion, and intracellular signalling [19-21]. Several lines of evidence suggest that changes in actin filament organisation play an important role in macrophage motility, adherence to surfaces and phagocytosis. Cellular dysfunctions associated with the cytoskeleton can cause retarded phagocytosis [22] and impaired phagosome-lysosome fusion [23], which may result in a diminished cellular killing and clearance of particles and pathogens from the lung.

The pro-inflammatory cytokine interleukin 1 (IL-1) is not normally produced by the cells of healthy individuals, exceptions being skin keratinocytes, some epithelial cells and some cells of the central nervous system. In response to inflammatory stimuli, however, there is a dramatic increase in the production of IL-1 by macrophages and other cell types [24]. There are two distinct proteins, IL-1α and IL-1β which are the products of two distinct genes but which recognise the same cell surface receptors [25]. IL-1 possesses a wide variety of biological activities. As well as inducing its own synthesis, IL-1 stimulates the secretion of TNF-α and IL-6 from macrophages/monocytes [26,27]. Normal production of IL-1 is vital for host responses to injury and infection, while prolonged secretion has been linked with a number of pathological conditions [28,29].

Our hypothesis in this study was that PM_10 _particles produce cytokine release and cytokine gene expression in macrophages by a process which involves calcium signalling and reactive oxygen species (ROS). Furthermore, we hypothesise that other effects of PM_10_, such as alterations in the cytoskeleton, are also mediated via signalling processes involving both ROS and calcium.

## Materials and Methods

### Particle Characteristics

Collection of PM_10 _samples was co-ordinated by Casella Stanger, London, England. Particles were collected onto TEOM filters in Marylebone Road, London, a site which had particularly high levels of traffic and therefore high levels of primary, combustion-derived nanoparticles. Ultrafine carbon black (UfCB) was obtained from Degussa (Printex 90), the average particle size was 14 nm. The characteristics and details of UfCB particles have been published previously [30].

### Particle Quantification

A single PM_10 _filter was placed into a bijou bottle and 0.5 ml phosphate buffered saline (PBS) added. The bottle was vortexed for 4 minutes to remove the particles from the filter and the resulting suspension transferred to a clean bijou bottle. The mass of particles was assessed by densitometry. As standards, a series of dilutions of UfCB particles were made, ranging from 15.625 μg/ml to 1 mg/ml in saline, sonicated for 5 minutes, and 75 μl of each concentration was added into triplicate groups of wells in a 96-well plate. Seventy-five microlitres of PM_10 _sample were added into a separate triplicate group of wells. The samples and standards were then read on a plate reader at 340 nm and the mass of particles calculated from a linear regression of the UfCB standards.

### J774.A1 Cell Culture

The mouse macrophage cell line J774.A1 (a kind gift from Dr W Muller GSF, Gauting, Germany) was routinely cultured in RPMI medium (Sigma) containing 5% foetal calf serum (FCS) and Penicillin/Streptomycin. Cells were cultured until confluency was reached and then scraped from the surface of the flasks using a cell scraper. The cells were counted and adjusted to 5 × 10^5^/ml in RPMI plus 5% FCS. Sterile 10 mm glass cover slips were placed in each well of a 24-well plate and 1 ml of cell suspension added to each well. Cells were incubated at 37°C for 24 hours prior to particle treatment.

### Isolation of Human Peripheral Blood Mononuclear Cells

Human peripheral blood mononuclear cells were prepared according to the protocol of Dransfield et al, [31]. In brief, two separate volumes of 40 ml of blood were withdrawn from healthy consenting volunteers and transferred to 50 ml sterile Falcon tubes containing 4 ml of 3.8% sodium citrate solution. Tubes were gently inverted and centrifuged at 250 g for 20 minutes, the plasma removed from each tube and pooled without disturbing the cell pellet. Dextran (Pharmacia), prepared as a 6% solution in saline was warmed to 37°C, before adding to the cell pellet (2.5 ml/10 ml cell pellet) and the volume made up to 50 ml with sterile saline. Tubes were gently mixed and the cells allowed to sediment at room temperature for 30 minutes. In order to prepare autologous serum, calcium chloride solution (220 μl 1 M/10 ml), was gently mixed with the plasma and incubated in a glass tube at 37°C until the clot retracted. Percoll (Pharmacia) gradients were made from a stock solution of 90% (18 ml Percoll + 2 ml 10x PBS, (Life Technologies, Paisley) without calcium or magnesium) to give final concentrations of 81%, 70% and 55% using 1x PBS. The separating gradient was prepared by layering 2.5 ml of 70% percoll over 2.5 ml 81% percoll. The leukocyte-rich fraction from the dextran sedimentation was transferred to sterile falcon tubes, 0.9% saline added to give a final volume of 50 ml and the tubes centrifuged at 250 g for 6 minutes. The pellet was resuspended in 55% percoll and 2.5 ml layered over the previously prepared separating gradients. Tubes were centrifuged at 290 g for 20 minutes and the mononuclear cells collected from the 55/70 layer. Cells were washed twice with PBS, counted, and resuspended in RPMI medium at a concentration of 5 × 10^6 ^cells/ml and 1 ml added to each well of a 24 well plate. For calcium imaging, cells were also set up in 6-well plates containing a 26 mm diameter sterile glass coverslip. The cells were incubated for 1 hour at 37°C, the medium removed and replaced with RPMI plus 10% autologous serum and incubated for 48 hours at 37°C. After the second incubation, the medium was replaced and the cells incubated for a further 72 hours prior to treatment.

### Cell Treatments

PM_10 _particles were diluted to give a final concentrations ranging from 5 μg/ml to 40 μg/ml in RPMI medium without serum and the suspension was sonicated for 5 minutes to disperse the particles. Cells which had been set up as described above, were washed twice with sterile PBS and 250 μl of particle suspension added to appropriate wells. UfCB particles were quantified as described for the PM_10 _and set up in parallel with PM_10 _particles at similar mass concentrations with J774 cells to investigate TNF-α release. One well received medium only (-ve control) and one received 250 μl of 1 μg/ml LPS (+ve control). The calcium antagonists were added concomitantly with the particles to give final concentrations of verapamil (100 μM), BAPTA-AM (50 μM), W-7 (250 μM), trolox (25 μM), and nacystelyn (5 mM). The cells were then incubated at 37°C for 4 hours and the supernatants removed and stored at -80°C until required. The cells cultured on 10 mm cover slips were fixed by the addition of 3% formaldehyde.

### J774.A1 Intracellular Calcium Measurements

J774.A1 cells were cultured and removed from flasks as described above. Cells were pooled into a single tube, adjusted to 4.5 × 10^6 ^cells/ml in RPMI plus 10% FCS and incubated at 37°C until required for the assay. One millilitre of cell suspension was transferred to an Eppendorf tube, centrifuged at 145 g for 2 minutes, the medium removed, the cell pellet resuspended in 1 ml PBS and again centrifuged at 145 g for 2 minutes. The PBS was removed and cells resuspended in serum-free RPMI medium containing 23 mM Hepes buffer. Cells were loaded with 1 μg/μl Fura 2-AM (Sigma) in DMSO, 2 μl/ml cell suspension, the tube wrapped in foil and incubated in a shaking water bath for 20 minutes at 34°C. After incubation, the tube was centrifuged at 145 g for 2 minutes at 4°C, the medium removed and replaced with 1.5 ml fresh RPMI without serum. The Fura 2-AM-loaded cells were transferred to a quartz cuvette with stirrer and placed immediately into a fluorimeter with heated block and basal fluorescence measurements obtained over a 100 second period. The fluorimeter was set up with to give excitation wavelengths of 340 nm and 380 nm, emission 510 nm and excitation and emission slit widths set at 5 nm. During the experiments, the cuvette temperature was kept constant at 37°C. After 100 seconds, 10 μl appropriate treatment in RPMI medium was added to the cuvette and the experiment allowed to run for a further 1700 seconds. Treatments consisted of PM_10 _to give a final concentration of 10 μg/ml with and without the calcium antagonists at the concentrations described above. Twenty microlitres of 5% Triton solution were added to the cuvette to lyse the cells to give the maximum fluorescence (Rmax) and the experiment continued for 500 seconds. To give the minimum fluorescence value (Rmin), 15 μl of 0.5 M EGTA in 3 M Tris buffer were added to the cuvette. The experiment was terminated after a further 500 seconds. The ratio of the fluorescence measurements at excitation wavelengths of 340 and 380 nm were converted to calcium concentration values using the method of Grynkiewicz et al, [32].

### Human and mouse TNF-α ELISA

The supernatants previously prepared were assayed for TNF-α protein content using a commercially available human TNF-α kit (Biosource) or mouse TNF-α kit (R&D Systems) according to the manufacturer's instructions. Briefly, each well of a 96-well plate was coated overnight with capture antibody, before washing with PBS containing 0.05% tween, and then adding test supernatant to the appropriate wells in triplicate groups. After incubation for 2 hours at room temperature, the wells were washed, a detection antibody added and incubated for a further hour at room temperature. The wells were then washed with PBS/tween before addition of Horseradish peroxidase (HRP)-conjugated streptavidin and incubated for 45 minutes at room temperature. Finally, the colour was developed by adding peroxidase substrate to each well, before reading the absorbance at 450 nm using a Dynatec plate reader.

### mRNA Extraction

The experiments described above were also used to generate cells for total RNA extraction. After removal of the supernatant, 400 μl Tri reagent (Sigma) was added to each well. The lysed cells were then scraped from the surface of the plate using a cell scraper and transferred to Eppendorf tubes. Two hundred microlitres of chloroform were added to each Eppendorf, vortexed for 15 seconds and allowed to stand at room temperature for 15 minutes. The resulting mixture was centrifuged at 12000 g for 15 minutes at 4°C. The colourless upper phase was transferred to a fresh Eppendorf, before adding 450 μl isopropanol. The mixed samples were allowed to stand for a further 10 minutes at room temperature. Again the tubes were centrifuged at 12000 g for 10 minutes at 4°C, the supernatant removed and the RNA pellet washed in 1 ml of 75% ethanol. The resulting samples were then vortexed briefly, centrifuged at 7500 g for 5 minutes at 4°C and the RNA pellet air-dried for 10 minutes. The RNA was then suspended in 50 μl diethylpyrocarbonate (DEPC)-treated water and stored at -70°C until required for quantification and Reverse Transcriptase-Polymerase Chain Reaction (RT-PCR).

### RT-PCR

The RT-PCR procedure was carried out using the Promega Access Kit. Briefly, a master mix of the kit reagents was prepared according to the manufacturers instructions. Ten microlitres of RNA at 0.03 μg/ml was added to 40 μl of the master mix, containing 10 μl of the appropriate human primers Glyceraldehyde Phosphate Dehydrogenase (GAPDH) or IL-1-α (MWG AG Biotech, Ebersberg, Germany). Tubes were placed in a thermal cycler which was programmed for the following temperatures and times. Following an initial 45 minute incubation at 48°, samples were cycled as follows: 94°C for 2 minutes, 95°C for 30 seconds, 60°C for 1 minute, 68°C for 2 minutes. This cycle was repeated 25 times for GAPDH and 30 times for IL-1 alpha. To conclude, the sample was incubated at 68°C for 7 minutes and then cooled to 4°C. The resulting RT-PCR products were separated by electrophoresis using a 2% agarose gel containing 1 μg/ml ethidium bromide and viewed under UV light. The RT-PCR bands were quantified by densitometry using Syngene software and the IL-1α band intensity was expressed as a percentage of the corresponding GAPDH band. These results were then expressed as a percentage of the untreated control.

### Calcium Imaging

Human mononuclear cells were isolated from blood as previously described followed by adhesion onto 26 mm glass coverslips contained in 6-well plates. Cells were seeded in RPMI medium containing 0.1% BSA and penicillin/streptomycin at a density of 5 × 10^5 ^cells/ ml and incubated at 37°C, 5% CO_2 _for 1 hour before washing with 1 ml PBS. Prior to particle treatment and digital enhanced video microscopy (Roper scientific), cells were loaded with the calcium-sensitive dye, Fura 2-AM (2 μg/ml in RPMI) (Sigma) for 30 minutes at 37°C. The coverslips were then washed with PBS, assembled into the microscope holder and 400 μl RPMI medium without phenol red (Sigma) added. The fluorescence ratio was observed (excitation 340 and 380 nm, emission 510 nm) at a magnification of 63× (Zeiss Axiovert microscope). Images were captured every 2 seconds by a Coolsnap fx Photometrics (Roper Scientific) camera controlled by Metafluor software. After 100 seconds particle treatment was added to the cells (100 μl of a 250 μg/ml stock solution of particles to give a final concentration of 50 μg/ml) contained in phenol red-free RPMI medium.

### F-Actin Staining

The cells cultured on cover slips and fixed with formaldehyde were washed three times with PBS and permeablised with 0.1%Triton for 4 minutes. Cells were then washed three times with PBS and the F-actin stained using 33 ng/ml Phalloidin-FITC (Sigma) in PBS for 30 minutes at room temperature. Cells were washed three times with PBS before staining with propidium iodide (10 μg/ml in PBS) for 5 seconds. Cells were further washed three times with PBS before being mounted on glass slides using Citifluor mounting medium. The cells were then examined using an Axiofluor fluorescence microscope. Images were captured and quantified using Metamorph software (Universal Imaging Corporation). Seven fields of view were captured from each treatment and the images deconvolved using the image software. The staining intensity of each cell was then measured using the analysis software.

### Statistical Analysis

Data from all of the experiments were analysed using analysis of variance with Tukey or Fishers multiple comparison test. Significance was set at p < 0.05.

## Results

### Intracellular Calcium Concentration in PM_10_-Treated J774.A1 Cells

**Figure 1:**
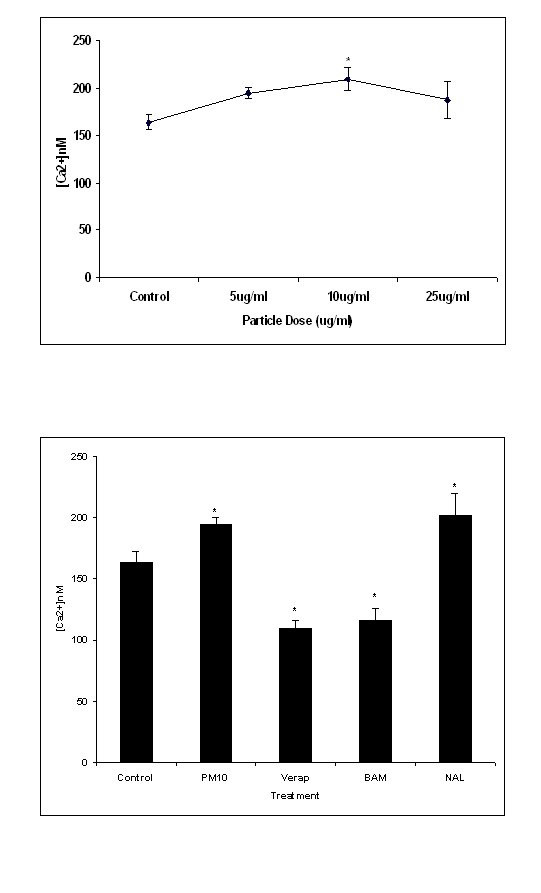
The cytosolic calcium concentration (nM) in J774 cells on treatment with 5–25 μg/ml PM_10 _particles for 1700 seconds (a) and with 10 μg/ml PM_10 _particles plus calcium antagonists (b). There was a significant difference only at the 10 μg/ml particle dose from the control (p < 0.05). With calcium antagonist treatment, there was a significant difference between all of the treatments and the control (p < 0.05). Data represents the mean ± SEM of the intracellular calcium concentration (nM). (n = 3).

The effects of PM_10 _on J774 murine macrophages was investigated at final concentrations of 5, 10 or 25 μg/ml PM_10_. Treatment of the cells with these particles produced a dose-dependent increase in cytosolic calcium concentration [Ca^2+^]_c _up to a concentration of 10 μg/ml. At 25 μg/ml the [Ca^2+^]_c _decreased to a value similar to the 5 μg/ml particle concentration. (Figure 1a). Subsequent treatment with thapsigargin to release the endoplasmic reticulum calcium store produced a further increase in cytosolic Ca^2+ ^indicating that the cells remained viable and confirming previous studies [33]. There was a statistically significant difference between control and PM_10 _treatments at 10 μg/ml (p < 0.05). The [Ca^2+^]_c _following concomitant treatment of cells with particles and calcium antagonists was reduced (figure 1b). Both the calcium channel blocker verapamil, and the calcium chelator BAPTA-AM significantly reduced (p,0.05) the intracellular calcium compared with PM_10 _alone. In contrast, the antioxidant Nacystelyn did not significantly reduce the PM_10_-stimulated [Ca^2+^] increase, with Ca^2+ ^concentration remaining significantly greater than the control value (p < 0.05). In our previous studies [34,35] we demonstrated that the antagonists used at the same concentrations used here caused no toxic effects to cells and that the drug treatment produced similar results to the untreated controls.

### Intracellular Calcium Concentration in PM_10_-Treated Human Monocytes

**Figure 2:**
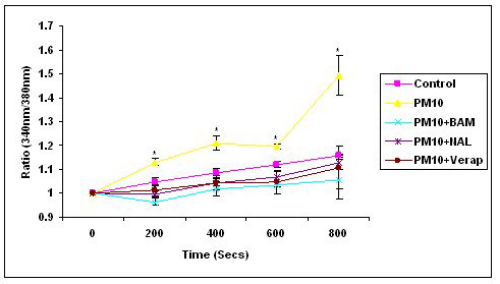
The intracellular calcium concentration (nM) in human monocytes on treatment with PM_10 _at a concentration of 10 μg/ml. Particles and calcium antagonists were added at zero time and the experiment run for 800 seconds in total (first 800 seconds shown). There was a significant difference between PM_10_-treated cells and PM_10 _and calcium antagonist treatment at each time point tested (p < 0.05). Data represents the mean ± SEM of the ratio of 340/380 nm. (n = 3).

PM_10 _also induced a significant increase in cytosolic calcium in the primary human monocytes (p < 0.05). The dose of 10 μg/ml final concentration was chosen as the dose at which a significant increase in [Ca^2+^]_c _had previously been observed (figure 1a). At time points from 600 to 800 seconds after the addition of particle/antagonist treatment, there was a rapid increase in [Ca^2+^]_c _with particles alone compared with the antagonists. In contrast to the antagonist treatment effects reported in figure 1, the antioxidant nacystelyn significantly inhibited the [Ca^2+^]_c _changes induced in the human monocytes treated with 10 μg/ml PM_10 _(figure 2). Both the intracellular calcium chelator BAPTA-AM and the calcium channel blocker verapamil also significantly inhibited the [Ca^2+^]_c _rise compared with particles alone (p < 0.05).

### Effect of UfCB and PM_10 _particles on TNF-α release by J774 cells

**Figure 3:**
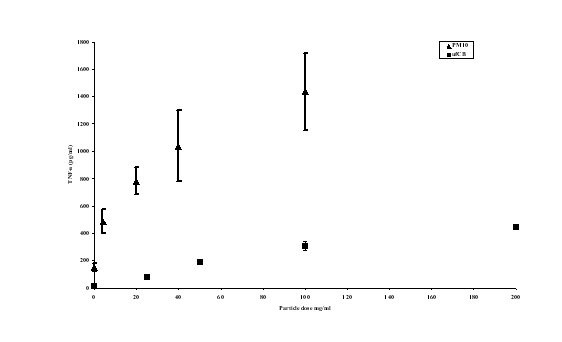
TNF-α protein release in J774 cells treated with UfCB or PM_10 _for 4 hours. There was significantly more TNF-α release in PM_10 _treated cells compared with an equal mass of UfCB. Data represents the mean ± SEM pg/ml TNF-α release (n = 3).

A comparison of the gram for gram dose effect of PM_10 _and UfCB particles on TNF-α release by J774 macrophages is shown in figure 3. The data show that PM_10 _particles caused significantly more TNF-α release as the same mass of UfCB particles by the mouse macrophage cell line.

### TNF-α release in PM_10_-treated Human Monocytes

**Figure 4:**
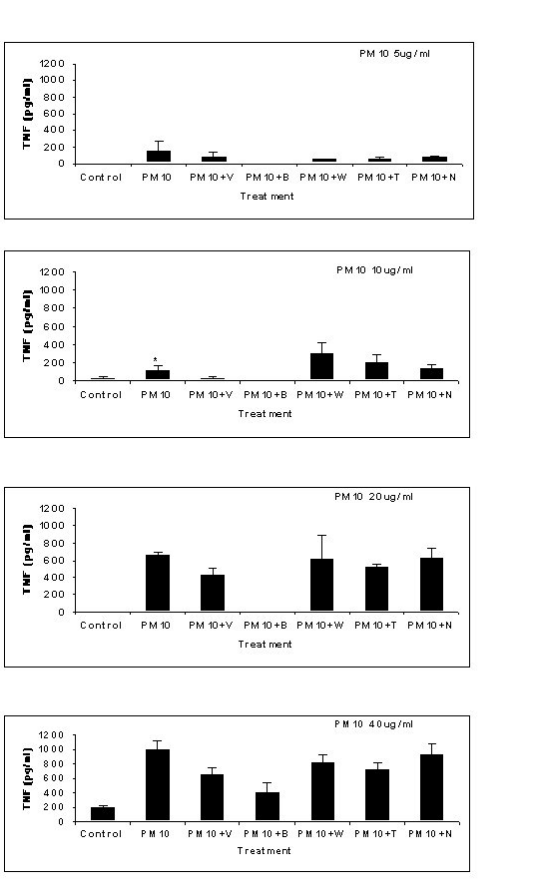
TNF-α release by human monocytes after treatment with PM_10 _(5–40 μg/ml) and with concomitant treatment with calcium antagonists for 4 hours verapamil (V), BAPTA-AM (B), W-7 (W), trolox (T), and nacystelyn (N). There was a significant difference between the untreated control and PM_10 _treatment only for the 10 μg/ml dose (p < 0.05). Data represents the mean ± SEM pg/ml TNF-α release (n = 5).

The release of TNF-α protein by human monocytes after treatment with varying concentrations of PM_10 _is shown in figure 4. The dose of particles ranged from 5 μg/ml to 40 μg/ml with concomitant treatment with calcium antagonists. There was a clear dose response of particle treatment from 10 μg/ml to 40 μg/ml. Within this range, the TNF-α concentration was approximately 170 pg/ml to 1000 pg/ml. At higher particle doses, the calcium antagonists reduced TNF-α release only marginally with the most dramatic and significant effect being seen with a particle concentration of 10 μg/ml with verapamil (V) and BAPTA-AM (B) treatments which reduced TNF-α release to 29 pg/ml and 7 pg/ml respectively (p < 0.05). There was no reduction in PM_10 _induced TNF-α release with W-7 (W), Trolox (T) or Nacystelyn (N) at any particle dose tested.

### IL-1 mRNA Expression

**Figure 5:**
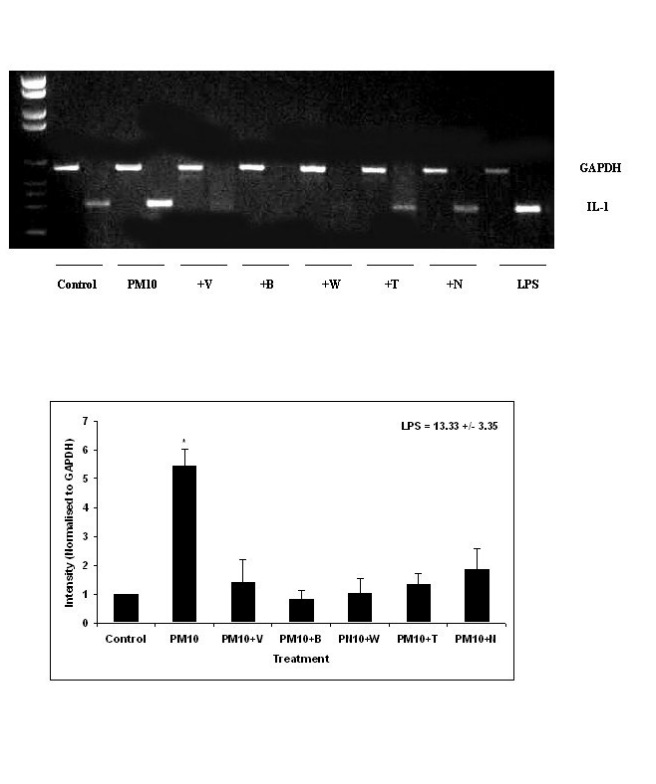
IL-1α mRNA expression in human monocytes treated with 10 μg/ml PM_10 _particles with and without calcium antagonists for 4 hours. The top panel shows a typical gel. The graph shows the IL-1α expression as a percentage of the GAPDH and normalised to the control. There was significantly greater expression of IL-1α mRNA in the PM_10 _treatment which was reduced to control levels with calcium antagonist treatment. Data represents the mean ± SEM of the mRNA intensity. (n = 3).

Treatment of human peripheral blood monocytes with 10 μg/ml PM_10 _for 4 hours produced a significant increase in IL-1α mRNA content compared with unstimulated cells (p < 0.05) (figure 5). The IL-1α band intensities were expressed as a percentage of the GAPDH band intensities and then normalised to the unstimulated control. PM_10 _induced a five fold increase in IL-1α mRNA expression compared with the control and on treatment with the calcium antagonists, this was reduced to values similar to the control. There was a significant difference between the PM_10 _exposed cells and concomitant treatment with all of the calcium antagonists and antioxidants tested (p < 0.05).

### F-Actin Staining

**Figure 6:**
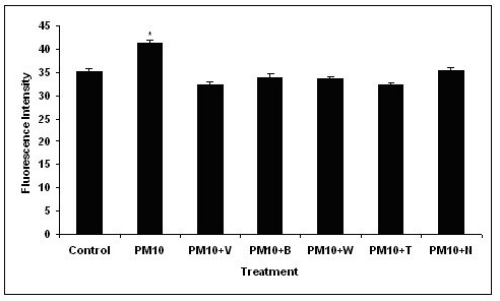
The fluorescence intensity of F-actin stained J774 cells after 10 μg/ml PM_10 _treatment and with calcium antagonist treatment for 4 hours. There was a significant difference in the intensity of PM_10_-treated cells compared with the untreated control (p < 0.05). There was no significant difference between the control and any other treatment. Data represents the mean ± SEM of the fluorescence intensity of the cells. (n = 3).

The fluorescence intensity of cells stained for F-actin after treatment with PM_10 _particles and calcium antagonists is shown in figure 6. Particles alone significantly increased the phalloidin-FITC fluorescence and hence the F-actin content of the cells compared with untreated cells. All of the calcium antagonists tested inhibited the PM_10 _induced increase in F-actin intensity to control levels and this was significantly different from particle only treatment (p < 0.05), although the increase in the fluorescence intensity of the PM_10_-treated cells was modest (a 5% difference).

## Discussion

There is evidence that increases in particulate air pollution correlate with increased morbidity and mortality from respiratory and cardiovascular causes [1,36-38] and the pro-inflammatory effects of PM_10 _are considered to drive these effects [39,40]. The present study aimed to investigate the effect of PM_10 _particles on oxidative stress- and calcium-related cytokine regulation in human monocytes and on the cytoskeleton in mouse J774 cells.

We have previously shown that ultrafine or nanoparticles enhanced the calcium influx into cells of a monocytic cell line (MM6) [19,34] and that these [Ca^2+^]_c _changes lead to production of the proinflammatory cytokine TNF-α [35]. We demonstrate here using calcium imaging, that PM_10 _particles can also stimulate entry of extracellular calcium into both J774 macrophages and human macrophage derived monocytes, and that this process is inhibited by a calcium channel blocker suggesting that the PM_10_, in a similar fashion to UfCB induces opening of plasma membrane calcium channels leading to a calcium influx. The results obtained using the antioxidant nacystelyn were confusing. In the J774 macrophages nacystelyn was unable to inhibit PM_10 _induced increases in cytosolic calcium concentration, whereas the same antioxidant was very effective in the human monocyte derived macrophages. This difference could be due to a species difference or a comparison between a cell line and primary cells. A number of cell lines have been demonstrated to exhibit aberrant calcium signalling pathways. Our previous studies using human macrophages suggest that ultrafine particle-induced increases in cytosolic calcium can be mediated by ROS [35] and since a large proportion of the particles within PM_10 _are ultrafine, it is conceivable that much of the calcium increase is ROS mediated, at least in part. However, PM_10 _also contains other substances, such as metals, that could influence this pathway. Metals would in fact be expected to increase the ROS production by the PM_10 _particles [41].

The present study clearly shows that the same dose of PM_10 _(10 μg/ml) that induces calcium elevation also stimulates significant increases in both TNF-α protein release and IL-1α mRNA production by macrophages. The calcium channel blocker verapamil and the intracellular calcium chelator BAPTA-AM reduced the calcium increase, TNF-α protein release and IL-1 mRNA expression by human monocytes when stimulated with PM_10 _particles. This is strong evidence to suggest that influx of extracellular calcium plays a key role in upregulating the proinflammatory response induced by PM_10 _that could lead to disease. However, the calmodulin inhibitor W-7 had little effect on TNF-α release, while it did inhibit IL-1 mRNA expression. The antioxidants also had variable abilities to block cytokine expression, inhibiting IL-1 mRNA production but not TNF-α protein release. These differences could be explained either by divergent pathways controlling expression of the two cytokines, or that TNF-α protein was measured in comparison to IL-1 mRNA. However, clearly both calcium and ROS are important in the regulation of IL-1α mRNA expression while only calcium is important in controlling TNF-α expression in macrophages exposed to PM_10_.

These studies indicate that on an equal mass basis PM_10 _is far more potent that UfCB in terms of its ability to induce TNF-α protein release. This is likely to be due to other components, such as metals and organic compounds other than the carbon core, within PM_10 _that can promote inflammation. It is also possible that components such as the UF particles and metals could interact to enhance toxicity as has been shown for ROS production in vitro and inflammation in vivro [41]. Our previous studies have failed to detect LPS in the PM_10 _particles, therefore it is unlikely that cytokine release, changes in intracellular calcium, and IL-1α gene expression can be explained solely by endotoxin.

As explained previously, the cytoskeleton is the scaffold of cells, and in the case of motile cells such as macrophages it is responsible for controlling movement. Disruption of the cytoskeleton, particularly via oxidative stress, is thought to disrupt cellular structure and hence function [42]. We have previously demonstrated that PM_10 _generates ROS [43]. The ability of the antioxidants trolox and nacystelyn to prevent the PM_10 _induced increase in F-actin staining in this study demonstrates that particle-derived ROS impact on the macrophage cytoskeleton. Our previous studies also demonstrate that Uf particle-induced ROS play a role in elevating the cytosolic calcium concentration of macrophages leading to increased TNF-α production [35]. The results of this study also suggest that both calcium signalling and ROS are important in modulating the F-actin cytoskeleton in response to PM_10 _exposure. As has been shown by other workers [44,45], in both the macrophage cell line and primary cells that F-actin is distributed as microfilaments around the cell, with special prominence at the leading edge of the cells. The microtubules in contrast are situated throughout the cell. Microtubules and actin filaments have previously been studied as targets of antitumour drugs [46] which mainly work by acting on microtubules and alter the dynamics of actin filaments. Changes in the distribution of actin filaments and their expression compared with normal cells may indicate alterations in the phagocytic ability of macrophages which may eventually lead to impaired particle clearance from the lungs. We show here that treatment of macrophages with PM_10 _particles increased the F-actin fluorescence signal in cells stained with FITC-labelled phalloidin, although changes in the distribution of actin filaments was not apparent from microscope analysis there appeared to be more cortical staining. In accord with the role of calcium and ROS in the induction of IL-1 expression, both of these factors appeared to play an important role in modulating the F-actin cytoskeleton.

The present study has shown that PM_10 _particles may exert their increased pro-inflammatory effects by modulating intracellular calcium signalling in macrophages leading to expression of proinflammatory cytokines. An additional consideration is the effects of particles on the cytoskeleton of the cell. Impaired cellular motility and phagocytic ability is likely to play a key role in particle clearance from the lung, thus perpetuating the effects of PM_10_. The role of calcium and ROS in other cellular responses are under investigation.
